# Novel strategy of combined interstitial macrophage depletion with intravenous targeted therapy to ameliorate pulmonary fibrosis

**DOI:** 10.1016/j.mtbio.2023.100653

**Published:** 2023-05-02

**Authors:** Zhongxian Li, Qiang Zhang, Jiawei Xiang, Mingyuan Zhao, Yuan Meng, Xuhao Hu, Tingting Li, Yifeng Nie, Huizhen Sun, Tun Yan, Zhuo Ao, Dong Han

**Affiliations:** a CAS Center for Excellence in Nanoscience, National Center for Nanoscience and Technology, Beijing, 100190, China; b Hebei Key Lab of Nano-biotechnology, Hebei Key Lab of Applied Chemistry, Yanshan University, Qinhuangdao, 066004, China; c University of Chinese Academy of Sciences, Beijing, 100049, China; d College of Life Sciences,Bejing University of Chinese Medicine, Beijing, 100029, China

**Keywords:** Combined strategy, Intervaginal space injection, Macrophage depletion, Pulmonary fibrosis, Targeted therapy

## Abstract

Idiopathic pulmonary fibrosis (IPF) is a severe interstitial lung disease with poor prognosis and high mortality rate. In the process of IPF, inflammatory dysregulation of macrophages and massive fibroblast aggregation and proliferation destroy alveoli, which cause pulmonary dysfunction, and ultimately lead to death due to respiratory failure. In the treatment of IPF, crossing biological barriers and delivering drugs to lung interstitium are the major challenges. In order to avoid the side effect of macrophages proliferation, we proposed, designed, and evaluated the strategy which combined macrophage depletion by intervaginal space injection and intravenous targeted therapy on bleomycin mouse model. We found that it inhibited pulmonary macrophages, reduced macrophage depletion in non-target organs, improved pulmonary drug targeting, impeded the progression of pulmonary fibrosis, and accelerated the recovery of pulmonary function. This combination therapeutic strategy shows good biosafety and efficacy, induces a targeted response, and is promising as a practical new clinical approach towards the treatment of pulmonary fibrosis.

## Graphical abstract

**Figure:**
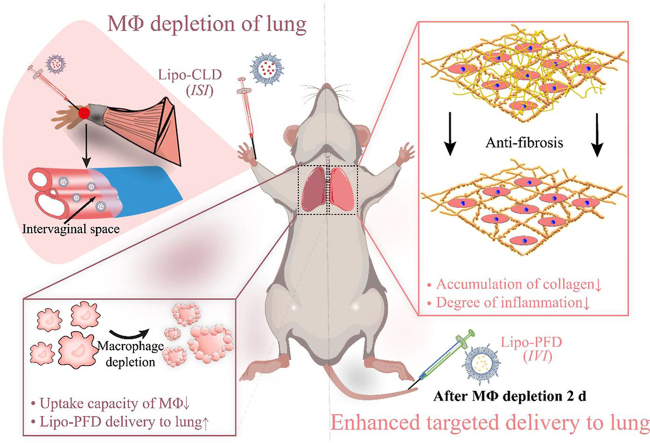

## 1 Introduction

Idiopathic pulmonary fibrosis (IPF) is an irreversible and fatal interstitial lung disease caused by genetic abnormalities, autoimmunity, trauma, occupational exposure, COVID-19, and other factors [1]. It is characterized mainly by fibroblast proliferation and aggregation, excessive extracellular matrix (ECM) precipitation in the alveoli and the lung interstitium, a reduction in lung volume, and thickening of the alveolar septum. Inflammation and scar formation then disrupt the alveolar structure, block gas exchange, cause pulmonary dysfunction, and ultimately lead to respiratory failure and even death [2]. IPF has poor prognosis, high mortality rate, and annually increasing incidence. Post-diagnosis survival is 3–5 years [3]. The pathogenesis of IPF is unclear and only pirfenidone and nintedanib are currently approved by the FDA as antifibrotic drugs. Nevertheless, they have narrow indications, limited duration of administration, and side effects [4]. Therefore, more efficacious treatment methods are urgently required for IPF.

Macrophages are vital components of the innate immune system. They have high plasticity and their homeostatic dysregulation is associated with atherosclerosis, cancers, fibrosis, and so on. Hence, they are potential therapeutic targets [5]. In the lungs, macrophages present antigens and play crucial roles in the occurrence and development of pulmonary fibrosis [6]. The latter is regarded as essentially a type of uncontrolled wound-healing response. In the earliest stages after tissue injury, epithelial or endothelial cells release proinflammatory mediators that initiate the antifibrinolytic-coagulation cascade which triggers coagulation of the temporary matrix and recruits macrophages to the injury site [7]. There, the latter undergo distinct phenotypic and functional changes (M1/M2) so they can regulate inflammation, tissue repair, regeneration, and regression [8]. Macrophages produce various cytokines and chemokines, which trigger the proliferation, differentiation and activation of fibroblasts and epithelial cells, endothelial cells and stem cells, thus affecting tissue repair [9,10]. During the later stages of repair, macrophages exhibit an anti-inflammatory phenotype that suppresses tissue damage and favors the restoration of normal tissue architecture [11]. Failure to control this process effectively results in persistent inflammation, defective repair mechanisms, and fibrosis [12]. Macrophages drive disease occurrence and regulate homeostasis. Thus, therapeutic strategies targeting macrophages have attracted research attention. Cell depletion strategies are efficacious immunotherapeutic methods for tumors, liver fibrosis, rheumatoid arthritis, and other diseases [[13], [14], [15]]. In pulmonary fibrosis, macrophage depletion reduces collagen deposition and impedes disease progress [16,17]. Thus, macrophage depletion is potentially effective in pulmonary fibrosis therapy. Though macrophage depletion is reversible, short-term immunodeficiency may lead to homeostatic imbalance. For this reason, macrophage depletion therapy should target specific organs to reduce the risk of systemic macrophage depletion.

Intervaginal space injection (*ISI*) is proposed as a new injection method for interstitial therapy, which uses the tarsal canal or carpal canal as the injection site to inject drugs into the interstitial tissue. Unlike traditional intravenous injection, *ISI* transports drugs to disease targets, which bypasses circulation. The interstitial system is potentially an efficient communication pathway and drug delivery route [[18], [19], [20], [21]]. It envelops all organs and tissues and plays vital roles in tissue structure and function, morphogenesis, and signal transduction. It also acts as a transport pathway for cancer cells, immunocytes, and microorganisms and participates in various pathophysiological processes including cancer cell metastasis, fibrosis, and edema [22,23]. Therefore, it is implicated in the occurrence and development of different diseases and can be involved in diagnostics and therapy. Studies have shown that during injury repair, the fascia is the main source of native cells in the wound including fibroblasts and inflammatory cells. The provisional matrix in the wound is derived from prefabricated matrix in the fascia and functions as a movable sealant pulling immunocytes and nerves upwards along the vasculature towards the wound site [24]. The exploitation of interstitial pathways to suppress inflammatory responses and excessive wound repair may be a breakthrough in pulmonary fibrosis treatment.

In the present study, we developed a novel combination treatment strategy known as “interstitial macrophage depletion-intravenous targeted therapy” in order to hinder the progression of pulmonary fibrosis and hasten the recovery of lung function. We prepared clodronate liposomes and injected them via *ISI* (upper limb) to deplete pulmonary macrophages. In this manner, we minimized damage to hepatic macrophages and improved the safety and targeted efficacy of macrophage depletion. We then administered liposome-encapsulated drugs to pulmonary fibrosis model mice via *IVI* (tail vein) and demonstrated that this treatment precisely targeted the lungs and was conducive to pathophysiological recovery. The combination treatment strategy was characterized by precise targeting and biosafety. Hence, it is potentially a safe and efficacious therapeutic option for pulmonary fibrosis.

## 2 Materials and methods

### 2.1 Materials

Clodronate disodium (CLD), 5-methyl-1-phenylpyridin-2(1H)-one (PFD), and bleomycin hydrochloride (BLM; 1.5–2.0 U/mg) were purchased from Macklin Biochemical Co. Ltd., Shanghai, China. DOTAP (1,2-dioleoyl-3-trimethylammonium-propane) was purchased from Corden Pharma Switzerland LLC, Liestal, Switzerland. D-Lin-MC3-DMA and 1,2-dimyristoyl-*rac*-*glycero*-3-methoxypolyethylene glycol-2000 (DMG-PEG2000) were purchased from AVT Pharmaceutical Tech Co. Ltd., Shanghai, China. Distearoyl-L-a-lecithin (DSPC) was purchased from Nippon Seika Co. Ltd., Nagaoka-shi, Niigata, Japan. Cholesterol was purchased from Avanti Polar Lipids, Inc., Alabaster, AL, USA. Bicinchoninic Acid Protein Assay, Cell Cycle and Apoptosis Analysis, Apoptosis and Necrosis Assay Kits, and DAPI (4’,6-diamidino-2-phenylindole) were purchased from Beyotime Institute of Biotechnology Co. Ltd., Shanghai, China. PKH67 Green Fluorescent Cell Linker Mini Kit was purchased from Sigma-Aldrich Corp., St. Louis, MO, USA. The 1,1-dioctadecyl-3,3,3,3-tetramethylindotricarbocyaineiodide (DiR) was purchased from Yeasen Biotechnology Co. Ltd., Shanghai, China. Sulfo-Cyanine5 (Cy5), Cell Counting Kit-8 (CCK-8), and a Hydroxyproline (Hyp) Assay Kit were purchased from Beijing Solarbio Science & Technology Co. Ltd., Beijing, China. Hoechst 33,342 and LysoTracker® Red were purchased from Thermo Fisher Scientific Inc., Waltham, MA, USA. A Sirius Red Total Collagen Detection Kit was purchased from Chondrex Inc., Woodinville, WA, USA. A Reactive Oxygen Species Assay Kit was purchased from BestBio Co. Ltd., Shanghai, China. *Anti*-TNF-α, *anti*-IL-1β, *anti*-TGF β1, anti-mouse CD16/32, *anti*-F4/80-PE, and anti-CD11b-FITC antibodies were purchased from BioLegend Inc., San Diego, CA, USA.

### 2.2 Cell culture and mice

The RAW264.7 murine macrophage and the L-929 murine fibroblast cell lines were obtained from the Cell Bank of the Chinese Academy of Sciences, Shanghai, China. The RAW264.7 macrophages were cultured in RPMI 1640 medium and the L-929 ​cells were cultured in Dulbecco's modified Eagle's medium (DMEM) supplemented with fetal bovine serum (FBS 10% v/v; Thermo Fisher Scientific) and antibiotic-antifungal solution (1% v/v; Thermo Fisher Scientific) at 37 ​°C and under a 5% CO_2_ atmosphere. All experiments were performed using RAW264.7 macrophages and L-929 ​cells in logarithmic growth phase. Eight-week-old male C57BL/6 mice were obtained from Beijing Vital River Laboratory Animal Technology Co. Ltd., Beijing, China. All animal handling procedures were conducted in accordance with the guidelines of the Ethics Committee of the National Center for Nanoscience, Beijing, China (approval number: NCNST21-2109-0407).

### 2.3 Preparation and characterization of Lipo-CLD

Liposomes loaded with CLD (Lipo-CLD) were prepared by the thin film hydration method, with slight modifications [30]. Briefly, the preparation conditions were modified to generate liposomes (Table S1A). Forty milligrams lipid (molar ratio of Dlin-MC3-DMA: DSPC: Chol: DMG-PEG: DSPE-PEG: DOTAP ​= ​10:2:7.7:0.3:80) was dissolved in chloroform and dried in a rotary evaporator at 45 ​°C to form a film. Then 1 ​mL of 20 ​mg/mL CLD was gradually added to the lipid film and the latter was emulsified under magnetic stirring for 30 ​min. Residual lipid film adhering to the bottle wall was hydrated with 7 ​mL normal saline for 5 ​min and homogenized by sonication (100% amplitude; 2/3-s pulse; on/off for 5 ​min) to obtain Lipo-CLD. Free CLD was removed using an ultrafiltration tube with molecular weight (MW) cutoff ​= ​100 kD. The range of the CLD: Lipo mass ratios was 1:1–1:10. Blank liposomes (without drug loading) and PFD-loaded liposome (Lipo-PFD) were prepared in the preceding manner and had the same lipid composition as Lipo-CLD. DiR-, PKH67-, and Cy5-loaded liposomes were prepared by mixing 1–2% DiR, PKH67, and Cy5, respectively, with Lipo and incubating at 4 ​°C. Unloaded dye was removed using an ultrafiltration tube with MW cutoff ​= ​100 kD.

The liposomes were uniformly dispersed by ultrasound and diluted to a certain concentration with deionized water. The hydrated particle sizes and surface charges of the liposomes were measured with a Zetasizer dynamic light scattering (DLS) instrument (Nano ZS90; Malvern Instruments, Malvern, UK). The diluted liposomes were dropped onto ultrathin carbon membranes, stained with 2% (w/v) uranyl acetate, dried at room temperature, and examined by transmission electron microscopy (TEM; Tecnai G2 F20 U-TWIN; FEI Co., Hillsboro, OR, USA). The CLD concentration was determined by ion chromatography. The Lipo-CLD was stored at 4 ​°C for 4 ​wks and its particle size and zeta potential were monitored at intervals. The Lipo-PFD was processed and analyzed in the same manner as the Lipo-CLD.

### 2.4 Evaluation of *in vitro* macrophage depletion

RAW264.7 macrophages were seeded in 96-well plates at a density of 10^4^/well and cultured for 24 ​h. The medium was discarded and the drug was diluted with fresh medium and replaced with those containing various concentrations of Blank liposomes, CLD, or Lipo-CLD. Each concentration was prepared in triplicate wells. After 24 ​h culture, the drug-containing media were discarded and the cells were washed thrice with phosphate-buffered saline (PBS). Then 100 ​μL of 10% CCK-8 medium was added to each well and the cells were maintained in a cell incubator for 4 ​h. A multimode microplate detection system was used to read the absorbances at 450 ​nm. RAW264.7 macrophages were cultured in six wells at a density of 5.0 ​× ​10^5^/well and co-incubated with PBS, CLD, or Lipo-CLD. In the latter two treatments, the CLD concentration was 20 ​μg/mL. The cell cycle ratios and apoptosis were measured by flow cytometry.

### 2.5 *In vivo* detection of macrophage depletion

Each C57BL/6 mouse was administered Lipo-CLD (equivalent to 0.5 ​mg CLD/mouse) in 200 ​μL normal saline by intervaginal space injection (*ISI*) or intravenous injection (*IVI*), respectively. After 48 ​h, the mice were sacrificed for perfusion and their livers and lungs were excised and sliced on a microtome to 20 ​μm thickness. CD68, F4/80, and terminal deoxynucleotidyl transferase dUTP nick end labeling (TUNEL) were used to stain the pulmonary macrophages, hepatic macrophages, and apoptotic cells, respectively. DAPI (300 ​mM, 5 ​min) was used to stain the nuclei. Lung sections were viewed and photographed under a confocal fluorescence microscope system fitted with the corresponding filter sets. The images were processed with ImageJ software (National Institutes of Health [NIH], Bethesda, MD, USA).

For flow cytometry, the lungs and livers were excised, homogenized, and digested and their cellular components were isolated. All operations were conducted according to the instructions of the reagent manufacturers. The Fc receptors were blocked, the cells were incubated with fluorescently labeled CD11b and F4/80, and the sample loading assay was performed in the flow cytometer.

### 2.6 Cellular effects of Lipo-PFD

*Cell uptake*. L929 cells were seeded into confocal dishes at a density of 10^4^/well. After 24 ​h incubation, the existing medium was replaced with fresh medium and 20 ​μL PKH67-loaded Lipo-PFD solution (10 ​μg/mL) was added. Incubation then proceeded for various lengths of time. PBS was then added to the wells to remove excess dye and the cells were stained with Hoechst 33,342 and LysoTracker® Red. Fluorescence images were immediately recorded under a fluorescence microscope (Ti–S; Nikon, Tokyo, Japan). Five randomly selected microscopic fields were quantified with ImageJ software.

*Cytotoxicity*. L929 cells were seeded in a 96-well plate at a density of 10^4^/well. After 24 ​h, PFD and Lipo-PFD media were separately added and cytotoxicity was assessed by adding complete medium containing 10% CCK-8. A multimode microplate detection system was used to read absorbances at 450 ​nm.

### 2.7 *In vivo* liposome distribution

C57BL/6 mice were injected by *ISI* and *IVI* at a dose equivalent to 0.5 ​mg CLD/mouse in 200 ​μL normal saline. After 48 ​h, DiR-labeled liposomes were then injected for subsequent imaging. The treatment groups were (1) untreated, (2) DiR dye, (3) DiR-labeled liposomes, (4) Lipo-CLD administered by *IVI* followed by DiR-labeled liposomes 48 ​h later, and (5) Lipo-CLD administered by *ISI* followed by DiR-labeled liposomes 48 ​h later. Six hours after DiR-labeled liposome injection, the major organs were excised for *in vitro* imaging. The distribution and intensity of the fluorescence of the various substances injected into the mice were evaluated with an IVIS spectral *in vivo* imaging system.

Mice were treated as previously described. They were injected with Cy5-labeled liposomes and their lungs were excised and sliced on a microtome to 20 ​μm thickness. The nuclei were stained with 300 ​mM DAPI for 5 ​min. The stained lung slices were then examined under a confocal fluorescence microscope fitted with the corresponding filter sets.

### 2.8 *In vivo* pulmonary fibrosis treatment

C57BL/6 male mice were anesthetized by continuous inhalation of 30% isoflurane gas mixture. A 2.5 ​mg/mL bleomycin (BLM) solution was prepared using normal saline. The neck skin of each mouse was dissected and the muscle tissue was separated to expose the trachea. BLM (5 ​mg/kg) was injected into each trachea. After 2 weeks, the mice were sacrificed and their lungs were excised, fixed in 4% (v/v) paraformaldehyde (PFA), and sectioned for hematoxylin-eosin (H&E) and Masson's trichrome staining.

The antifibrotic efficacies of PBS (negative control), PFD (positive control), Lipo-PFD, Lipo-CLD (*IVI*)+Lipo-PFD, and Lipo-CLD (*ISI*)+Lipo-PFD were assessed in a mouse pulmonary fibrosis model. The mice were treated with Lipo-CLD (2.5 ​mg/mL; 200 ​μL) on days 0 and 7. Lipo-PFD (10 ​mg/kg) was intravenously injected every 2 ​d after the first Lipo-CLD dose. As PFD is an orally administered clinical drug, the PFD group received it by gavage. Mouse body weights were measured after the start of the treatment and every 2 ​d thereafter.

After the final Lipo-PFD dose on day 16, the mice were placed in a spirometer, allowed to adapt in the cavity until their breathing was stable, and monitored for tidal volume (TV), airway resistance (RAW), respiratory rate, inspiratory time (T_i_), exhalation time (T_e_), and mid-expiratory tidal flow (EF50).

The mice were then euthanized and blood samples were collected. The blood was left to stand at room temperature for 2 ​h and centrifuged at 1500 ​rpm for 5 ​min. The sera were collected and subjected to biochemical indicator analyses. The lungs and livers were excised and weighed. Portions were frozen and later subjected to enzyme-linked immunosorbent assay (ELISA). The lungs, livers, and other organs were fixed with 4% (v/v) PFA, embedded in paraffin, and sectioned. The tissue slices were subjected to immunofluorescence, immunohistochemistry (IHC), H&E and Masson's trichrome staining. Pathological changes in each tissue section were observed under an optical microscope. The Ashcroft scores, semiquantitative alveolar air areas, and collagen content in the lung sections were also evaluated. The total collagen content and the Hyp levels in the lungs were detected using commercial ELISA kits following the manufacturer's instructions.

### 2.9 Statistical analysis

All data are means ​± ​standard deviation (SD). Statistical analysis was performed in Microsoft Excel (Microsoft Corp., Redmond, WA, USA) and GraphPad Prism v. 9.0 (GraphPad Software, La Jolla, CA, USA). Significant differences between treatment means were evaluated by non-parametric one-way analysis of variance (ANOVA). ∗P ​< ​0.05, ∗∗P ​< ​0.01, and ∗∗∗P ​< ​0.001.

## 3 Results

### 3.1 Clodronate liposome preparation and characterization

**Fig. 1:**
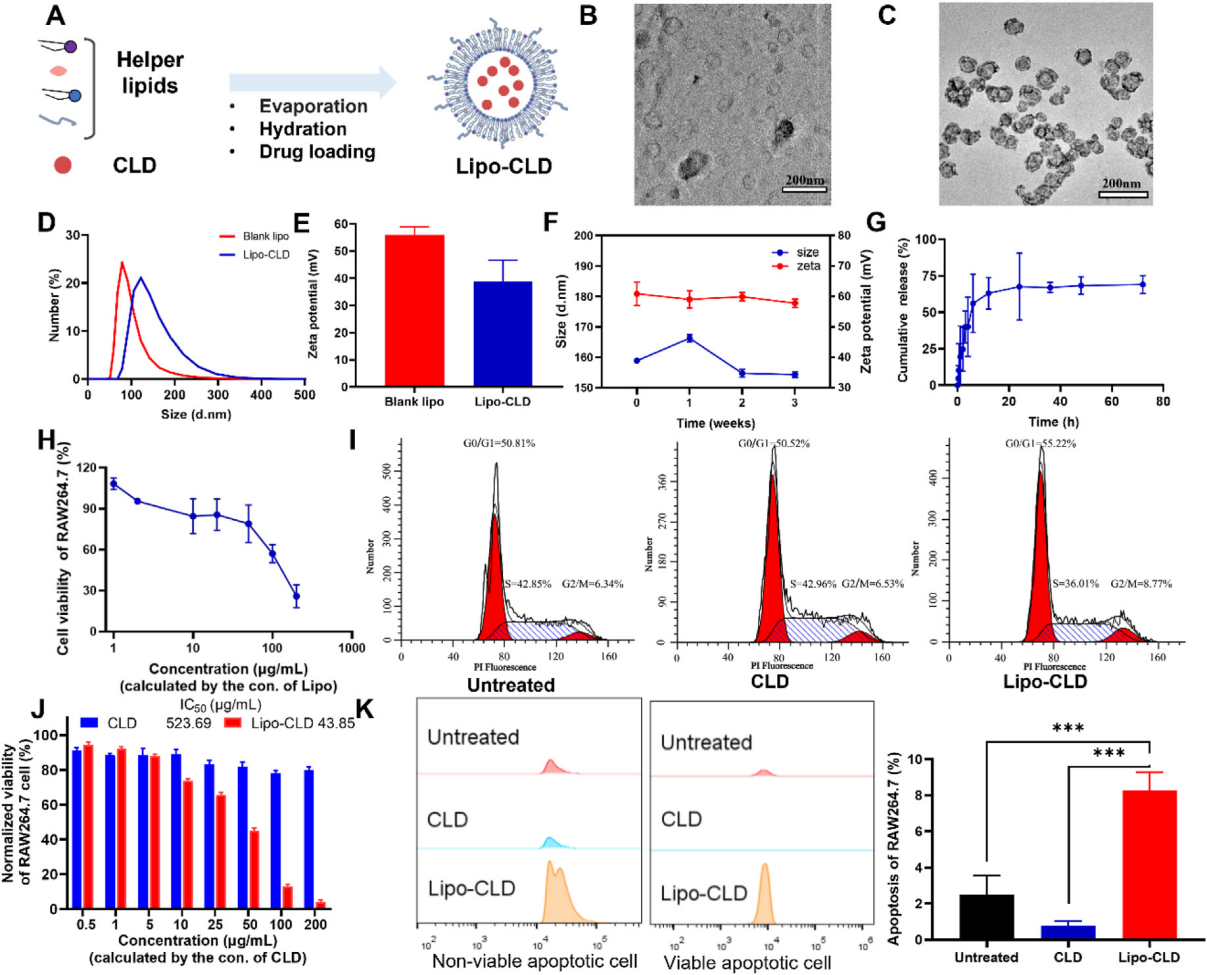
**Preparation and characterization of Lipo-CLD and evaluation of its macrophage-depleting effect *in vitro*.** (A) Schematic diagram of Lipo-CLD preparation. (B) TEM morphology of unloaded liposomes. (C) TEM morphology of Lipo-CLD. (D) DLS determination of liposome hydration particle size before and after drug loading. (E) DLS determination of liposome zeta potential before and after drug loading. (F) Changes in Lipo-CLD particle size and zeta potential after 3 ​wks. (G) *In vitro* Lipo-CLD release rate. (H) Evaluation of cytotoxicity of various liposome concentrations to RAW264.7 macrophages. (I) Flow cytometry and propidium iodide (PI) staining indicating effect of Lipo-CLD on cell cycle. (J) Relationship between CLD concentration and Lipo-CLD cytotoxicity. (K) Flow cytometry and Annexin V/PI staining identifying ability of Lipo-CLD to induce apoptosis in RAW264.7 macrophages.

Clodronate (CLD) is a routinely administered macrophage-depleting small-molecule drug with short half-life and poor lipid solubility [25]. To improve its bioavailability, we prepared Lipo-CLD by the thin film hydration method (Fig. 1A). Transmission electron microscopy (TEM) revealed that the liposomes were spherical both before and after drug encapsulation and had relatively uniform particle size and good dispersion (Fig. 1B and C). Dynamic light scattering (DLS) disclosed that the mean hydrated particle size and the average polydispersity index (PDI) of the unloaded CLD liposomes were ∼80 ​nm and 0.21, respectively. After drug loading, the average liposome particle size was 120 ​nm (Fig. 1D). The zeta potential of the drug-free liposomes was 55.8 ​± ​3.12 ​mV. After drug loading, the Lipo-CLD potential had decreased to 33.8 ​± ​7.9 ​mV because of the negative charge contributed by disodium chlorophosphate ionization in aqueous solution (Fig. 1E). We conducted hemolysis experiments to evaluate the biocompatibility of the liposomes and verified that they maintained the hemolysis rates at higher concentrations (Fig. S3). Lipo-CLD encapsulation efficiency and drug loading were satisfactory when the CLD: auxiliary lipid mass ratio was in the range of 1:2–1:5 (Fig. S1). The Lipo-CLD particle size and zeta potential remained nearly constant during storage at 4 ​°C for 3 ​wks. Hence, the liposomes were stable (Fig. 1F). In order to further evaluate the drug sustained release effect of the designed liposomes, a release rate was determined, and the release of CLD could still be detected after 48 ​h of sustained release *in vitro* (Fig. 1G).

### 3.2 *In vitro* evaluation of macrophage depletion by Lipo-CLD

CLD may be encapsulated in liposome phospholipid bilayers. As macrophages phagocytose the liposomes, the CLD is gradually released, accumulates in the macrophages, and causes apoptosis. We used the CCK-8 method to explore the cytotoxicity of blank liposomes to RAW264.7 macrophages. When the blank liposomes concentrations were <100 ​μg/mL, the cell survival rate was >70% after 24 ​h incubation. Hence, the blank liposomes had good biosafety (Fig. 1H). RAW264.7 macrophages were then incubated with various concentrations of both CLD and Lipo-CLD. As free CLD could not readily penetrate the cell membranes, its killing effect was weak. By contrast, Lipo-CLD demonstrated a far stronger killing effect (Fig. 1J). The IC50 values of Lipo-CLD and CLD on RAW264.7 macrophages were 43.85 ​μg/mL and 523.69 ​μg/mL, respectively. We then used flow cytometry to assess the ability of Lipo-CLD to induce apoptosis in RAW 264.7 macrophages. Propidium iodide (PI) staining showed that the RAW264.7 macrophages treated with free CLD presented no cell cycle anomalies compared to untreated RAW264.7 macrophages. By contrast, the RAW264.7 macrophages treated with Lipo-CLD-treated were blocked at G0/G1 (Fig. 1I). Annexin V/PI staining showed that the proportion of apoptotic cells was significantly higher in the Lipo-CLD than the untreated control group (Fig. 1K). Thus, the drug-loaded liposomes effectively depleted the macrophages.

### 3.3 *In vivo* macrophage depletion by Lipo-CLD

**Fig. 2:**
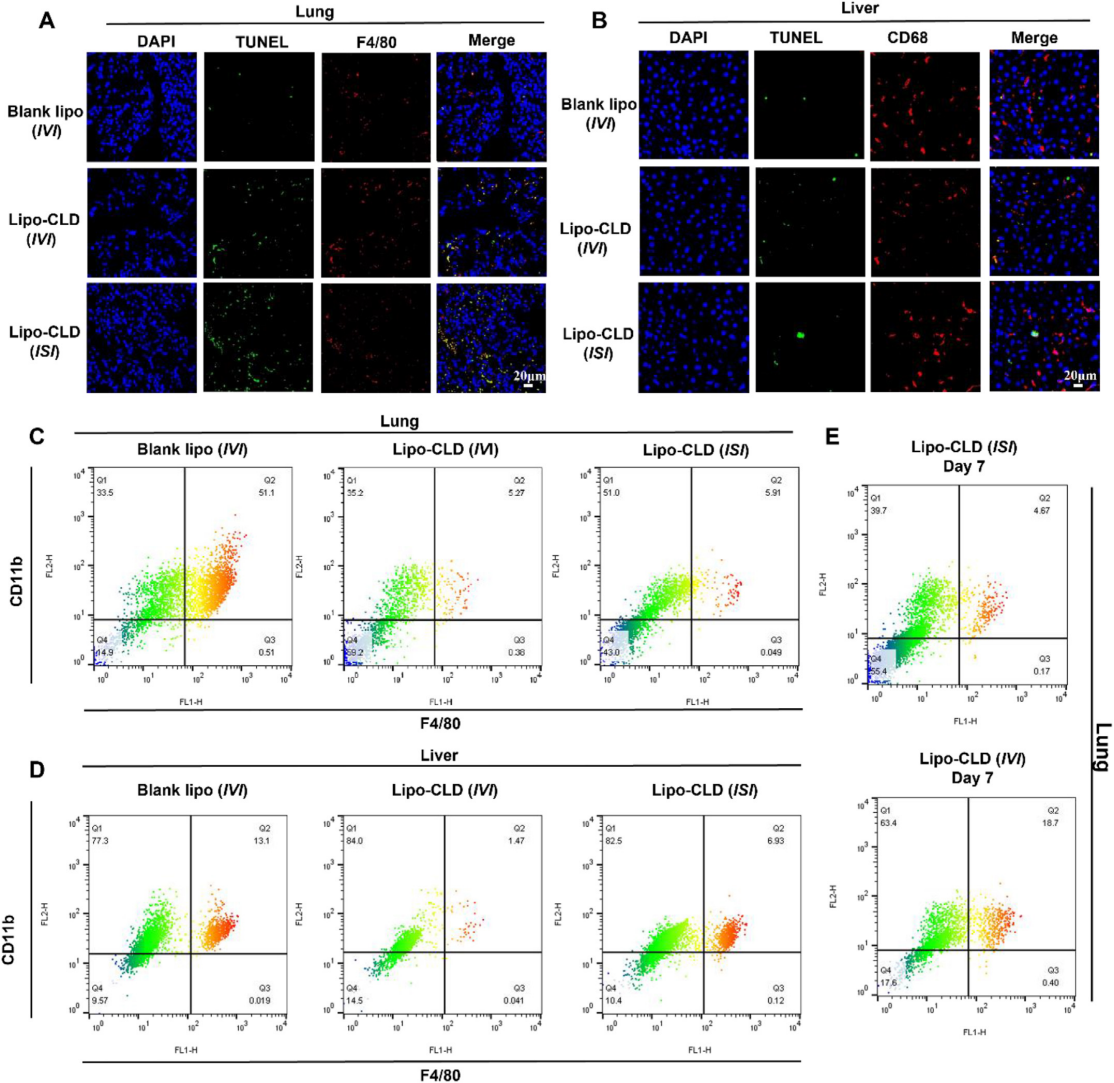
***In vivo* macrophage depleting by Lipo-CLD**. (A) Immunofluorescence staining of sections of lung tissue subjected to different Lipo-CLD injection methods. CD68-labeled macrophages (red); TUNEL-labeled apoptotic cells (green); 4′,6-diamidino-2-phenylindole (DAPI)-labeled nuclei (blue). (B) Immunofluorescence staining of liver tissue sections. CD68-labeled macrophages (red); TUNEL-labeled apoptotic cells (green); DAPI-labeled nuclei (blue). (C) Flow cytometry detection of pulmonary macrophages in lung tissue subjected to different Lipo-CLD injection methods. (D) Flow cytometry detection of hepatic macrophages in liver tissue subjected to different Lipo-CLD injection methods. (E) Flow cytometry detection of pulmonary macrophages on day 7 after depletion.

We opted for intervaginal space injection (*ISI*) rather than the conventional administration route to minimize perturbations of physiological functions and more effectively target pulmonary macrophages. We performed F4/80 and CD68 immunofluorescence staining to label lung and liver macrophages (red), respectively, and terminal deoxynucleotidyl transferase dUTP nick end labeling (TUNEL) staining to label apoptotic cells (green) (Fig. 2A and B). The Lipo-CLD treatment significantly decreased the number of pulmonary macrophages but increased the number of apoptotic cells relative to the control. The *ISI*-administered group displayed significantly more macrophage depletion than the control.

Both the mice administered Lipo-CLD by intravenous injection (*IVI*) and those administered Lipo-CLD by *ISI* exhibited significant pulmonary macrophage depletion. In the former case, however, CLD-loaded liposomes accumulated in the Kupffer cells and simultaneously depleted both hepatic and pulmonary macrophages. We then used CD11b and F4/80 to label the macrophages and subjected them to flow cytometry to verify their depletion in the lungs and liver (Fig. 2 and S5). Two days after *IVI* and *ISI* administration of Lipo-CLD, large amounts of pulmonary macrophages were depleted and there was no significant difference between Lipo-CLD injection methods (Fig. 2C). However, *ISI* administration of Lipo-CLD depleted hepatic macrophages to a lesser extent than *IVI* administration of Lipo-CLD (Fig. 2D). By day 7, there were still fewer depleted macrophages in response to *ISI* than *IVI* administration of Lipo-CLD (Fig. 2E).

### 3.4 Pirfenidone liposome preparation and cellular effects

**Fig. 3:**
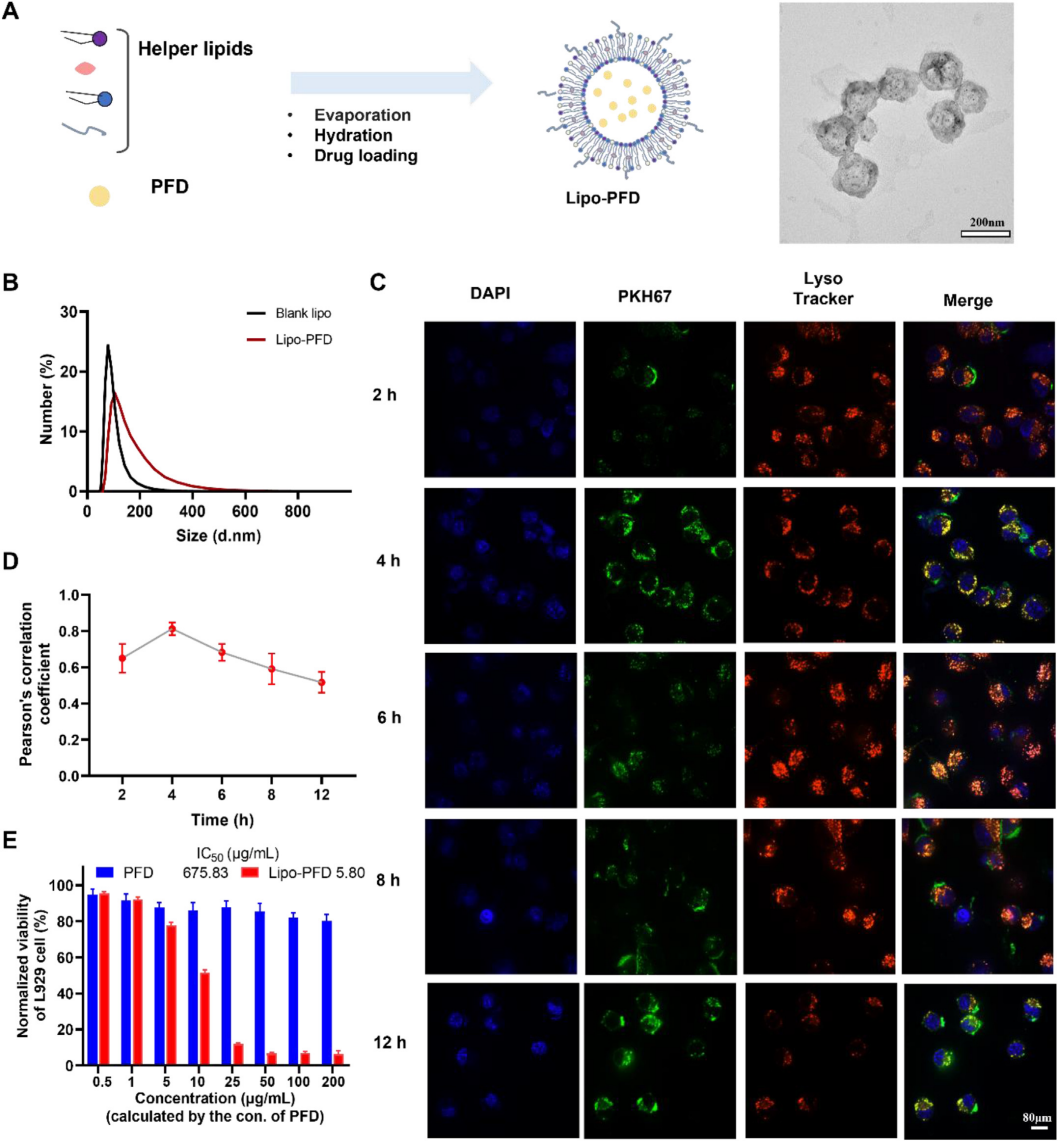
**Lipo-PFD preparation and its effects on L929 ​cells**. (A) Schematic diagram of Lipo-PFD preparation and TEM morphology. (B) DLS determination of hydration particle size for Lipo-PFD. (C) Fluorescence imaging of Lipo-PFD and L929 lysosome co-localization. PKH67: Lipo-PFD; LysoTracker® Red: lysosome; DAPI: nucleus; (D) Data analysis of Lipo-PFD and L929 lysosome co-localization. (E) Inhibition of L929 ​cell activity by various Lipo-PFD concentrations.

Pirfenidone (PFD) is a pyridone compound approved by the Food and Drug Administration (FDA) for the treatment of pulmonary fibrosis. It effectively prevents and reverses fibrosis and scar formation. As it is not targeted, however, its efficacy is limited. We selected PFD as a model drug, loaded it into liposomes (Lipo-PFD) (Fig. 3A), and validated the efficacy of Lipo-PFD in pulmonary fibrosis treatment and interstitial macrophage depletion. TEM disclosed that the liposomes were relatively uniform. DLS revealed that their average hydrated size and zeta potential were ∼105 ​nm (Fig. 3B) and ∼35.3 ​mV (Fig. S4), respectively.

We then labeled Lipo-PFD with PKH67 (green) and co-incubated it with L929 fibroblasts to determine its *in vitro* effects. Fluorescent staining was performed at various time points to observe Lipo-PFD capture by the L929 fibroblasts (Fig. 3C). A lysosomal probe localized intracellular lysosomes (red), the L929 ​cells abundantly captured the Lipo-PFD after prolonged incubation time, and Lipo-PFD co-localization with the lysosomes (yellow) peaked at ∼4 ​h. As the incubation time progressed, the liposomes induced the proton sponge effect and rupture in the lysosomes. Thence, PFD was released from the Lipo-PFD and colocalization decreased (Fig. 3D). We then used the CCK-8 assay to evaluate L929 inhibition by different Lipo-PFD concentrations (Fig. 3E). For all treatments, the number of viable cells decreased with increasing PFD dosage but the differences among concentrations were not significant. At 5 ​μg/mL, Lipo-PFD potently lowered L929 ​cell viability. The IC50 values for Lipo-PFD and PFD on the L929 ​cells were 5.80 ​μg/mL and 675.83 ​μg/mL, respectively.

### 3.5 *In vivo* liposome distribution in interstitial macrophage depletion-intravenous targeted therapy

**Fig. 4:**
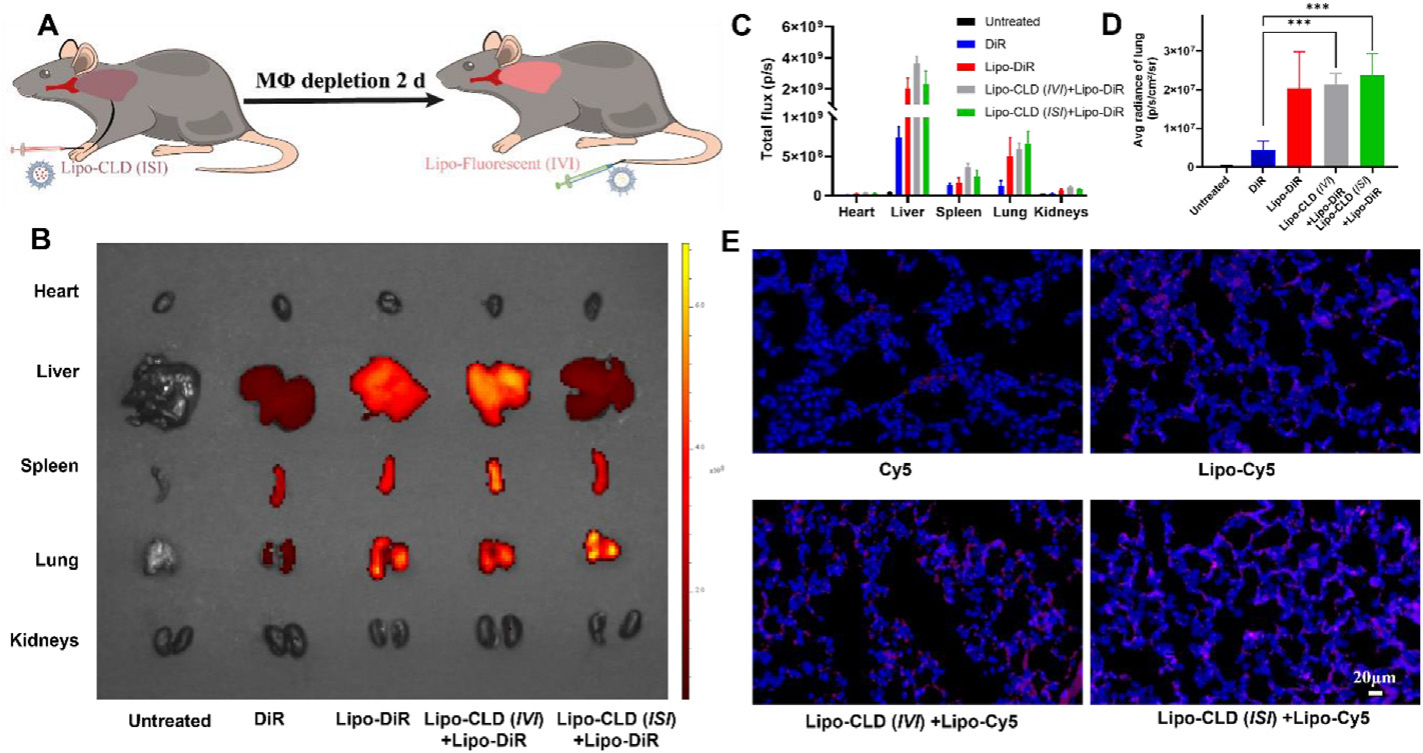
***In vivo* liposome distribution in response to the interstitial macrophage depletion-intravenous targeted therapy strategy.** (A) Schematic diagram of interstitial macrophage depletion-intravenous targeted therapy strategy. (B) Tissue distribution of DiR-labeled liposomes following different drug administration strategies. (C) Statistics of fluorescence intensity distributions of liposomes in organs subjected to different drug administration strategies. (D) Average fluorescence intensity of liposomes accumulated in lungs of mice subjected to different drug administration strategies. (E) Fluorescent tissue sections showing distribution of liposomes in lungs of mice subjected to different drug administration strategies.

The foregoing results indicated that Lipo-CLD administered by *ISI* provided enhanced pulmonary macrophage depletion but reduced hepatic macrophage depletion. Therefore, we proposed an interstitial macrophage depletion-intravenous targeted therapy strategy. After two days of macrophage depletion by Lipo-CLD administration via *ISI*, we administered dir-labeled liposomes by *IVI* to mice with pulmonary fibrosis (Fig. 4A). Another 6 ​h later, their organs were imaged *ex vivo*. We labeled the liposomes with DiR fluorescent dye to verify the targeting efficacy of the combination strategy and detect liposome distribution in various organs by the different injection methods (Fig. 4B). The pulmonary macrophages were depleted through Lipo-CLD. And 2 ​d later, the Liposomes labeled were injected by *IVI*. The combination strategy group (Lipo-CLD (*ISI*)+Lipo-DiR) showed relatively superior lung targeting and reduced hepatic aggregation following *ISI* depletion (Fig. 4C). The fluorescence signal intensity was 1.65-fold higher and 1.46-fold higher in the lungs of the Lipo-CLD (*ISI*)+Lipo-DiR group than in those of the non-depleted (Lipo-DiR) and tail vein depletion (Lipo-CLD (*IVI*)+Lipo-DiR) groups, respectively (Fig. 4D). Thus, depletion of lung macrophages by *ISI* administration promotes pulmonary targeting of the drug and greatly enhances drug delivery to the lung while reducing uptake and destruction of liposomes by other organs.

To demonstrate the specific distribution of liposomes in the lung after depletion of lung macrophages, we treated mice with Lipo-CLD for 24 ​h and then gave mice Cy5-labeled liposomes by the tail vein. After 6 ​h, the lung sections exhibited liposome accumulation and interstitial infiltration. The lung sections of the Lipo-CLD (*ISI*)+Lipo-Cy5 group displayed stronger fluorescence signals than those of the other groups. In this case, relatively more Lipo-Cy5 accumulated at the alveolar cell sites. Hence, pulmonary Lipo-Cy5 distribution was enhanced. The foregoing results indicated that the interstitial macrophage depletion-intravenous targeted therapy strategy for drug delivery promoted lung accumulation and interstitial penetration and suggested that this protocol holds promise for the treatment of pulmonary fibrosis.

### 3.6 The combination strategy ameliorates pulmonary fibrosis progression

**Fig. 5:**
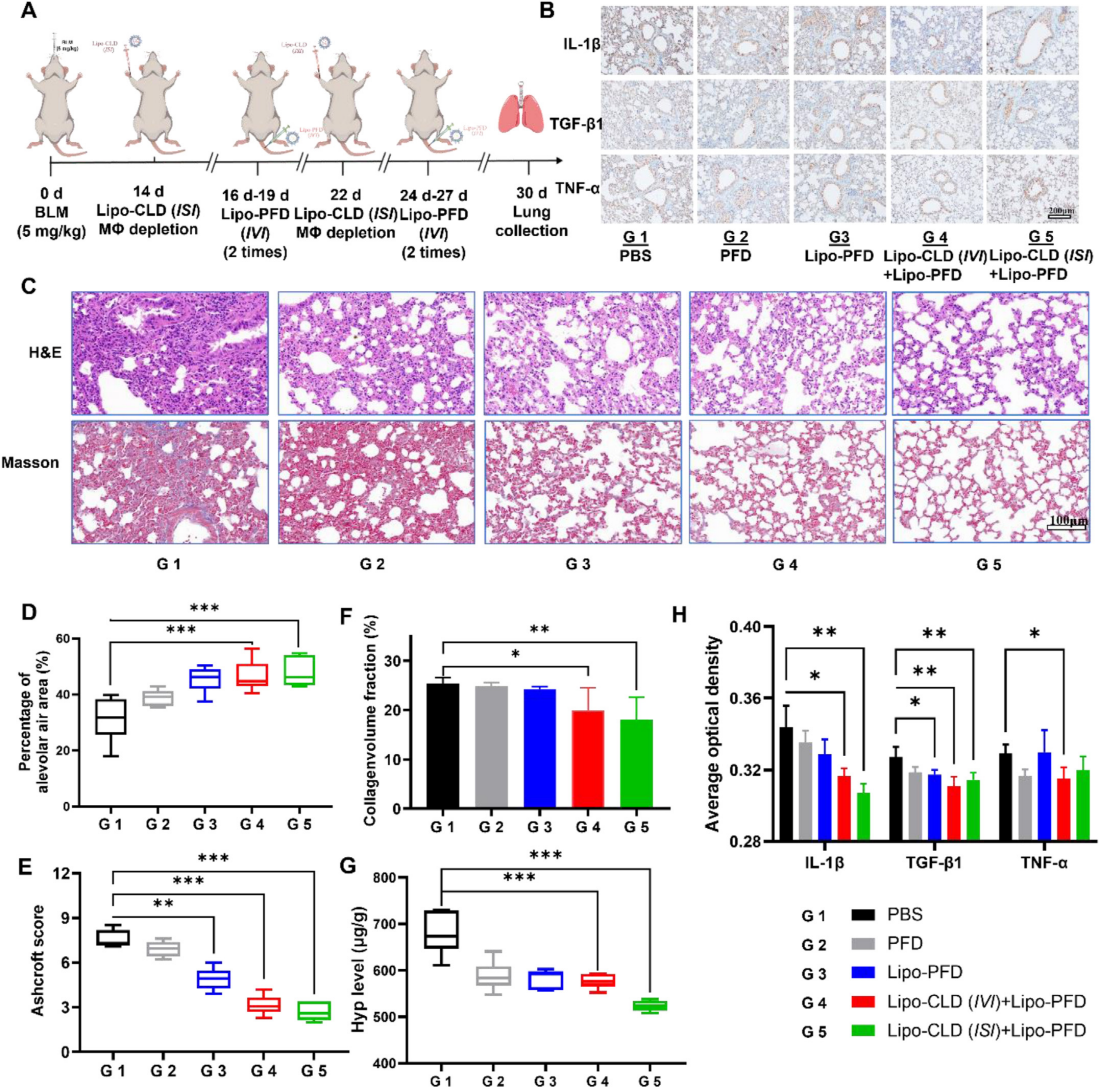
**Combination therapy strategy for pulmonary fibrosis.** (A) Schematic diagram of combination therapy strategy. (B) IHC sections of various drug administration strategies. (C) H&E and Masson's trichrome staining of different drug administration strategies. (D) Alveolar cavitation areas in different drug administration strategies. (E) H&E staining and Ashcroft scores. (F) Collagen deposition ratios according to Masson's trichrome staining. (G) Pulmonary Hyp levels in response to different drug administration strategies. (H) IHC optical density (OD) analysis.

*ISI*-mediated macrophage depletion favors lung targeting and interstitial liposome penetration. Therefore, the combination drug delivery strategy is a novel modality that improves the efficiency of delivering therapeutic drugs for pulmonary fibrosis. We established a pulmonary fibrosis model by tracheal bleomycin administration to verify the therapeutic efficacy of the combination strategy in pulmonary fibrosis. At 14 ​d after modeling, macrophages in the lungs were inhibited by *ISI* administration of Lipo-CLD. After 48 ​h of pulmonary macrophage depletion, Lipo-PFD was administered twice by *IVI* with a 3-d interval between injections. The combination treatment was repeated once (Fig. 5A). H&E and Masson's trichrome staining disclosed that after 14 ​d modeling, the alveolar walls and structure were thickened and damaged, respectively, and presented with prominent blue-purple collagen fibers (Fig. 5C). After two treatments, the Lipo-CLD (*ISI*)+Lipo-PFD mice (combination strategy group) exhibited significantly decreased alveolar wall areas and increased alveolar cavitation (Fig. 5D). The Ashcroft score and the collagen content according to Masson's trichrome staining were significantly decreased and the pathological assessment was significantly changed. Therefore, the severity of the fibrosis was substantially reduced and damage to the alveolar epithelial cells was mitigated (Fig. 5E and F). Compared with the other treatment modalities, the combination strategy significantly reduced the levels of the fibrosis biomarker Hyp. Thus, the combination strategy can reduce collagen deposition and impede the progression of pulmonary fibrosis (Fig. 5G).

Macrophages produce the anti-inflammatory cytokines TGF-β and TNF-α in response to external stimuli. IL-1β and TNF-α work synergistically to increase the number of myofibroblasts, thereby promoting pulmonary fibrosis progression. Immunohistochemical (IHC) analyses of fibrotic lung sections showed that the Lipo-CLD (*ISI*)+Lipo-PFD treatment significantly inhibited IL-1β (proinflammatory cytokine) secretion and downregulated TGF-β and TNF-α. For these reasons, the combination strategy impairs macrophage function and attenuates fibrotic tissue accumulation (Fig. 5B and H).

There were no significant differences in body weight among the groups during treatment (Fig. S6). The ratio of the lung weight to the body weight indicated that the lung coefficient was significantly lower in the treatment group than the model group. Therefore, the Lipo-CLD (*ISI*)+Lipo-PFD treatment had a positive effect on the lung coefficient. There were no significant differences among groups in terms of their blood alanine aminotransferase (ALT) or aspartate aminotransferase (AST) levels. Hence, neither CLD nor PFD significantly altered liver function (Fig. S7). By contrast, the combination treatment significantly reduced the alkaline phosphatase (ALP) and creatine phosphokinase (CK) levels. Thus, the Lipo-CLD (*ISI*)+Lipo-PFD combination treatment could ameliorate pulmonary infarction.

Compared with the untreated and oral drug groups, the Lipo-PFD treatment significantly improved lung function, airway resistance (RAW), and Ti/Te ratio (Fig. S8) and eventually ameliorated airway obstruction, bronchoconstriction, and compliance. Compared with the other treatments, Lipo-CLD (*ISI*)+Lipo-PFD significantly prolonged the tidal volume and increased the midexpiratory tidal flow (EF50). Hence, it alleviates persistent lung injury associated with fibrosis.

## 4 Discussion

Clodronate (CLD) is a first-generation bisphosphonate that is administered for the treatment of osteoporosis and osteolytic metastases [26]. As osteoclasts originate in the monocyte-macrophage system, CLD is used for macrophage depletion. However, it has a short half-life and cannot penetrate phospholipid bilayers or achieve effective cell killing. Liposomes encapsulating CLD are recognized and engulfed by hepatic Kupffer cells and macrophages are depleted through apoptosis [25]. The macrophage depletion effect of CLD is exploited mainly in antitumor therapy. The depletion of hepatic macrophages decreases liposome retention in the liver. In this manner, the liposomes may more effectively target tumor sites. Here, we constructed Lipo-CLD to deplete pulmonary macrophages and maximized CLD encapsulation efficiency by adjusting the ratios of the liposome components. The liposomes had uniform particle size as well as good dispersibility and stability. *In vitro* experiments demonstrated that Lipo-CLD strongly inhibited macrophage activity and augmented the macrophage depletion efficacy of CLD.

The macrophage-depleting action of CLD is nonspecific. Thus, CLD may also deplete systemic non-target macrophages. Therefore, it is crucial to modify CLD so that it targets only the macrophages at pulmonary fibrosis sites and does not disrupt those in non-target organs. To reach their target sites, liposomes must be able to overcome several obstacles including host immunity, the endothelial barrier, filtration, and the blood-brain barrier. The use of different injection methods that target various positions can lower the foregoing barriers and improve the diagnosis and treatment of diseases. Intravenously injected CLD liposomes accumulate in the liver and spleen. Subcutaneously injected CLD liposomes reach the lymph nodes. Intraperitoneally injected CLD liposomes deplete both peritoneal and mesenteric macrophages [[27], [28], [29]]. Pulmonary macrophages include the alveolar and pulmonary interstitial subtypes. The former resides in the alveoli while the latter occurs in the pulmonary interstitium. In pulmonary fibrosis, alveolar destruction, interstitial hyperplasia, and fibrous deposition hinder drugs from attaining the interstitial macrophages. However, there is highly efficient communication within the interstitial system. For this reason, we proposed a novel method of intrathecal space injection that could achieve highly efficacious targeted disease therapy. *In vivo* Lipo-CLD administration via *ISI* realized superior pulmonary macrophage depletion with minimal effect on the macrophages in other organs including the liver.

Pirfenidone (PFD) is a pyridine approved by the FDA as an antifibrotic agent for the clinical treatment of pulmonary fibrosis. Several studies confirmed that PFD inhibits α-SMA and type I collagen overexpression through the TGF-β1 pathway. However, oral PFD administration is associated with severe gastrointestinal distress, systemic photosensitivity reactions, poor patient tolerance, and short *in vivo* half-life (2.2 ​h). Hence, PFD has very limited practical clinical efficacy. Therefore, the objectives of the therapeutic development of PFD have included the enhancement of lung-targeted delivery efficiency, mitigation of non-target organ damage, amelioration of side effects, and prolongation of onset time. Here, we prepared Lipo-PFD to improve drug bioavailability. Lipo-PFD had uniform particle size and good dispersibility, sustained release effect, and stability.

Wound repair is divided into the coagulation, inflammatory, fibroblast migration/proliferation, and remodeling phases [7]. Secretion of inflammatory mediators by epithelial and endothelial cells initiates the antifibrinolytic-coagulation cascade which, in turn, triggers coagulation of the temporary matrix. Platelet aggregation and degranulation promote vasodilation, increase vascular permeability, and allow recruitment of neutrophils, macrophages, lymphocytes, and eosinophils to the injury site. These inflammatory cells produce various cytokines and chemokines that amplify the inflammatory response and trigger fibroblast proliferation. When fibroblasts are activated, they transform into myofibroblasts that express α-smooth muscle actin and secrete an extracellular matrix (ECM). During the final wound remodeling phase, myofibroblasts promote wound contraction, epithelial/endothelial cells divide and migrate to the temporary matrix, and damaged tissue is regenerated. Fibrosis occurs when wounds are severe, tissue-damaging stimuli persist, or the repair process is dysregulated. Both fibroblasts and macrophages play critical roles in the formation of pulmonary fibrosis. Therefore, we proposed a combination strategy of interstitial macrophage depletion-intravenous targeted therapy. Through *ISI*, this procedure initially targets macrophage activity in fibrotic lung tissues. Thence, PFD inhibits pulmonary fibroblasts. In this manner, this novel combination strategy impedes the progress of pulmonary fibrosis.

## 5 Conclusion

On the basis of the vital roles of macrophages and fibroblasts in the pathogenesis of pulmonary fibrosis, we proposed herein a combination strategy of interstitial macrophage depletion and intravenous targeted therapy. This treatment approach involves the depletion of pulmonary macrophages by interstitial Lipo-CLD injection followed by intravenous administration of antifibrotic Lipo-PFD. Interstitial macrophage depletion suppresses pulmonary inflammatory cells without harming non-target organs, thereby creating a buffer for the repair of fibrotic site lesions. The subsequent targeted intravenous administration enriches the drug in the lungs and accelerates lung function recovery. The combination strategy involves different injection methods for each therapeutic agent and is promising for the safe and efficacious targeted treatment of idiopathic pulmonary fibrosis and potentially other diseases as well.

## Credit author statement

DH and ZA designed the research. ZL, QZ and JX performed drug synthesis. ZL, QZ, MZ, YM, XH, TL, YN, HS and TY conducted the cell experiments and animal experiments, ZL and JX analyzed the data, ZL, JX, and QZ wrote the manuscript. All authors have read and approved the final submitted manuscript.

## Funding

This work was supported by the Key Research Program of 10.13039/501100002367CAS (QYKJZD-SSW-SLH02) and the Key Research Program of Frontier Sciences of 10.13039/501100002367CAS (ZDBS-LY-SLH036).

## Declaration of competing interest

The authors declare that they have no known competing financial interests or personal relationships that could have appeared to influence the work reported in this paper.

## Data Availability

Data will be made available on request.
