# Supplementary material for: Novel strategy of combined interstitial macrophage depletion with intravenous targeted therapy to ameliorate pulmonary fibrosis
Source: Mater Today Bio. 2023 May 2;20:100653. doi: 10.1016/j.mtbio.2023.100653 (PMC10192919; doi:10.1016/j.mtbio.2023.100653)
Supplement: Multimedia component 1 [file mmc1.docx]

**Supplementary Information**

**Novel strategy of combined interstitial macrophage depletion with intravenous targeted therapy to ameliorate pulmonary fibrosis**

Zhongxian Li^#^，Qiang Zhang^#^, Jiawei Xiang， Mingyuan Zhao，Yuan Meng，Xuhao Hu, Tingting Li, Yifeng Nie, Huizhen Sun, Tun Yan, Zhuo Ao *, Dong Han*


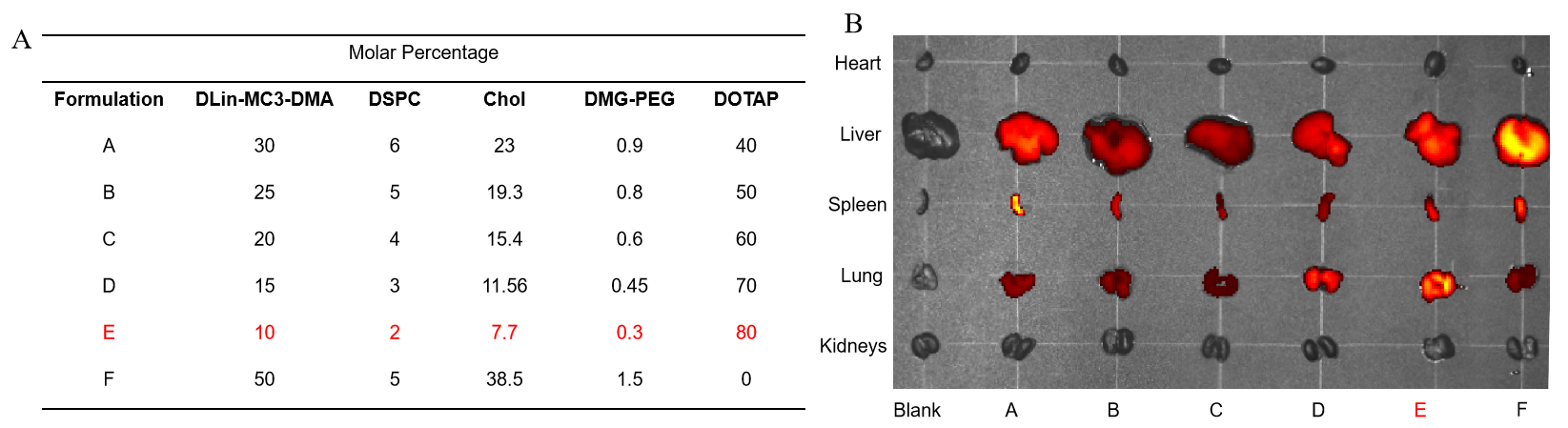


S1. Lipid composition ratio optimization (A) Molar percentage of lipid composition (B) Organ distribution of liposomes with different lipid compositions.


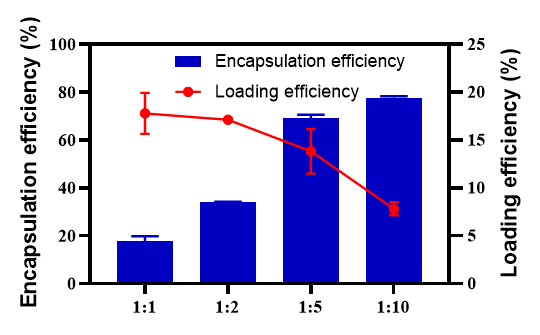


S2. Encapsulation efficiency and loading efficiency of different drug-to-lipid ratios.


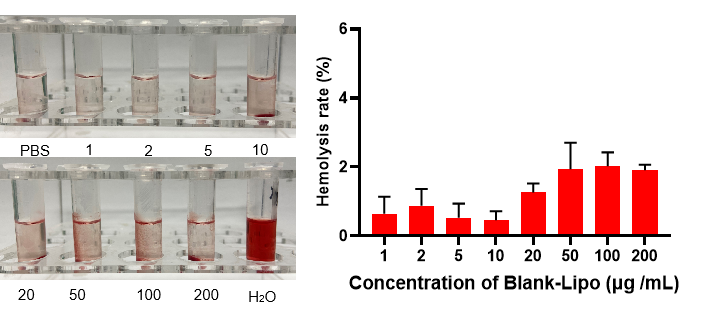


S3. Hemolysis rates of different liposome concentrations.


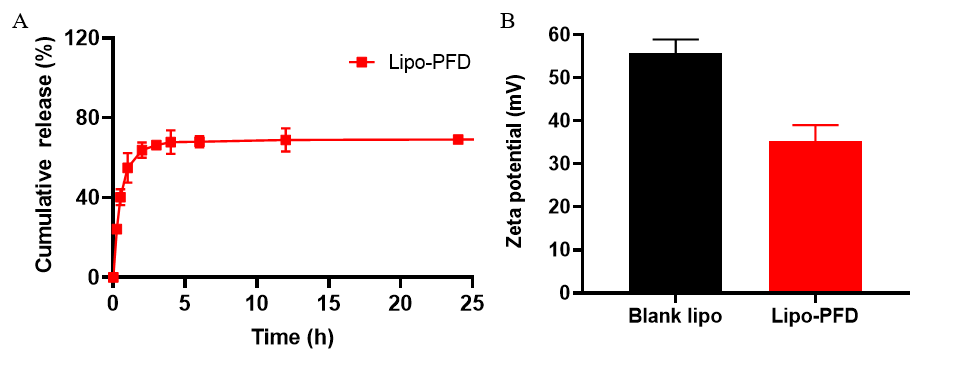


S4. Cumulative release rate and Zeta potential of Lipo-PFD.


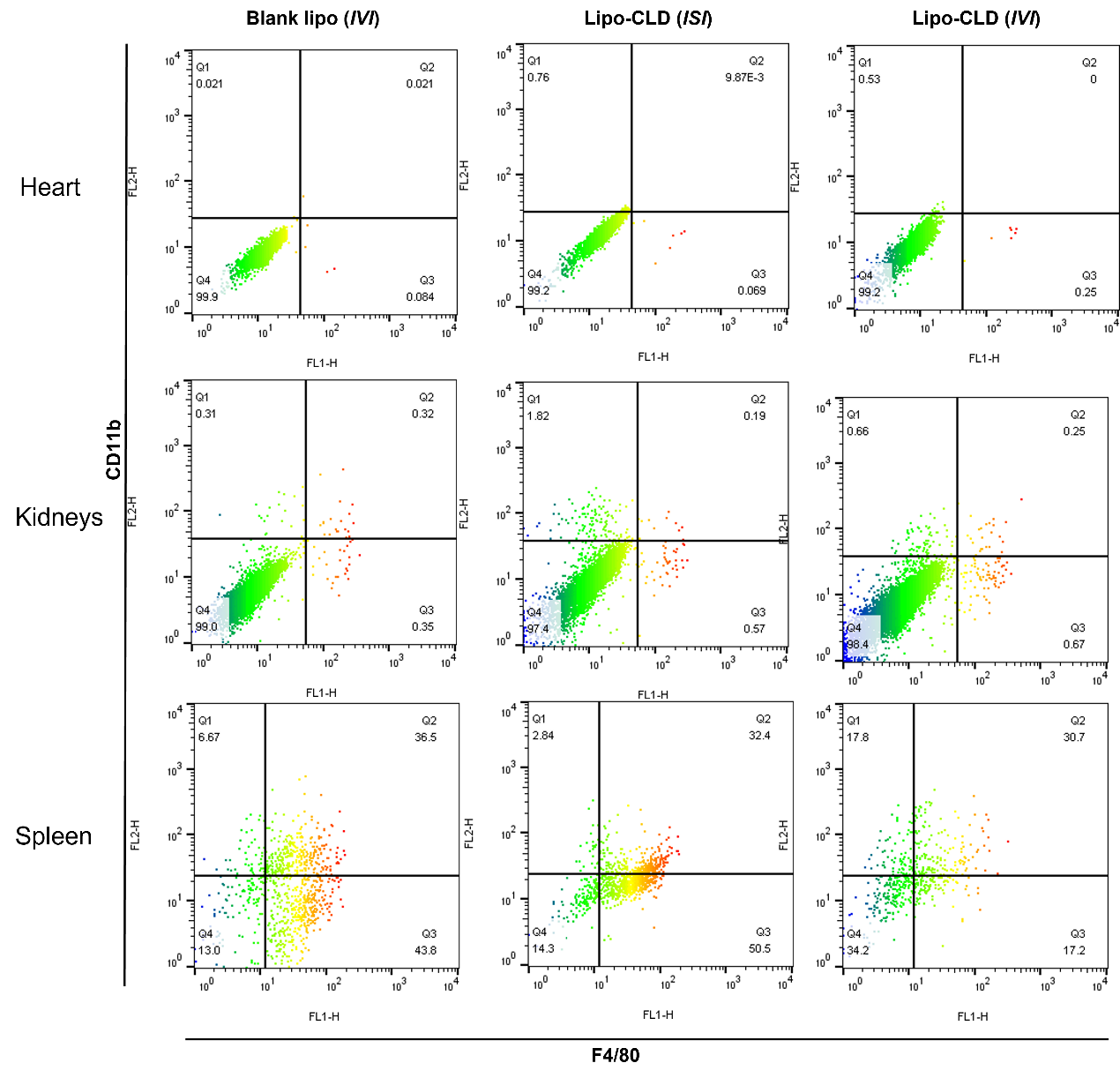


S5. Detection of macrophage depletion in different organs by flow cytometry.


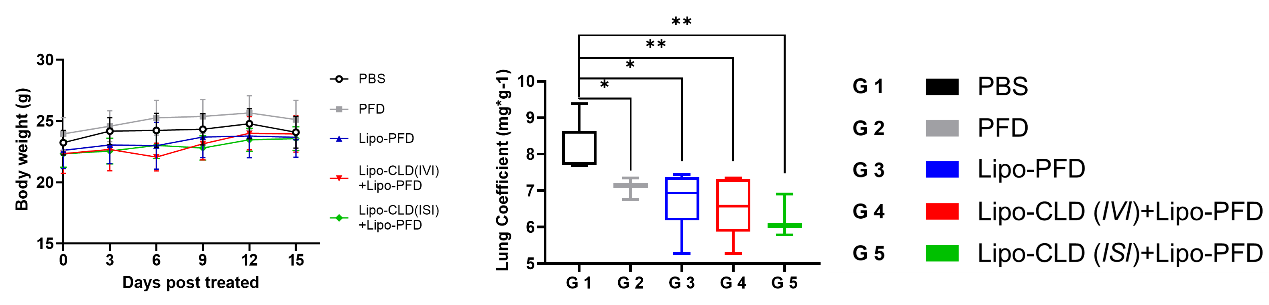


S6. The body weight and ratio coefficient of lung to its body weight of the mice during drug administration.


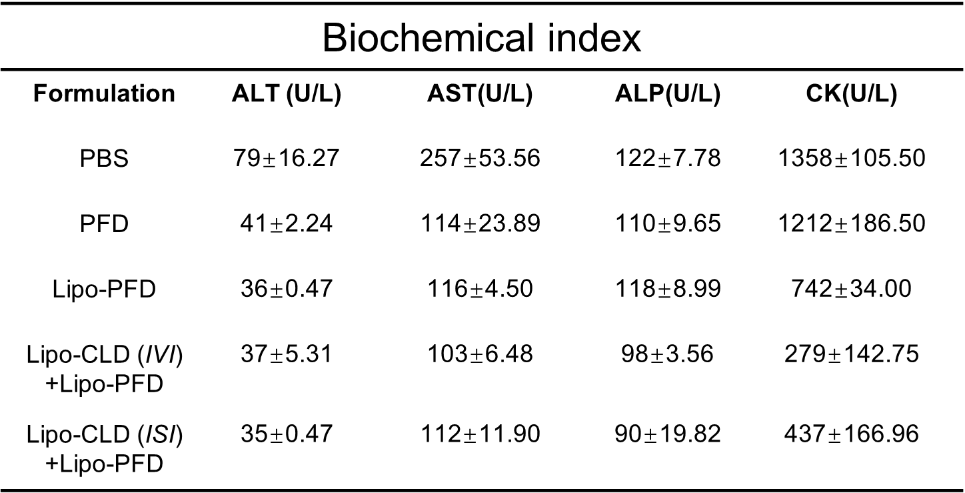


S7. Blood biochemical indicators before and after treatment. ALT: Alanine Aminotransferase; AST: Aspartate Aminotransferase; ALP: Alkaline Phosphatase; CK: Creatine Phosphokinase.


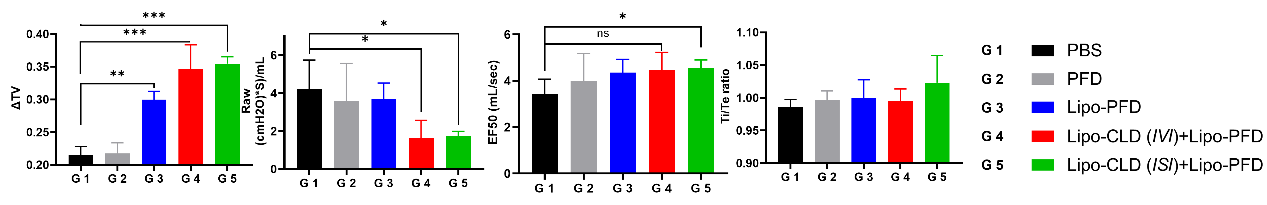


S8. Improved lung function after combined strategy treatment. RAW: Airway Resistance; EF50: Expiratory Flow 50; Ti: Inspiratory Time; Te: Expiration Time.
